# Leveraging Multilayered “Omics” Data for Atopic Dermatitis: A Road Map to Precision Medicine

**DOI:** 10.3389/fimmu.2018.02727

**Published:** 2018-12-12

**Authors:** Debajyoti Ghosh, Jonathan A. Bernstein, Gurjit K. Khurana Hershey, Marc E. Rothenberg, Tesfaye B. Mersha

**Affiliations:** ^1^Division of Immunology, Allergy & Rheumatology, Department of Internal Medicine, University of Cincinnati, Cincinnati, OH, United States; ^2^Division of Asthma Research, Department of Pediatrics, Cincinnati Children's Hospital Medical Center, University of Cincinnati, Cincinnati, OH, United States; ^3^Division of Allergy and Immunology, Department of Pediatrics, Cincinnati Children's Hospital Medical Center, University of Cincinnati, Cincinnati, OH, United States

**Keywords:** atopic dermatitis, omics, multi-omics integration, endotypes, biomarkers, bioinformatics, machine learning, network analysis

## Abstract

Atopic dermatitis (AD) is a complex multifactorial inflammatory skin disease that affects ~280 million people worldwide. About 85% of AD cases begin in childhood, a significant portion of which can persist into adulthood. Moreover, a typical progression of children with AD to food allergy, asthma or allergic rhinitis has been reported (“allergic march” or “atopic march”). AD comprises highly heterogeneous sub-phenotypes/endotypes resulting from complex interplay between intrinsic and extrinsic factors, such as environmental stimuli, and genetic factors regulating cutaneous functions (impaired barrier function, epidermal lipid, and protease abnormalities), immune functions and the microbiome. Though the roles of high-throughput “omics” integrations in defining endotypes are recognized, current analyses are primarily based on individual omics data and using binary clinical outcomes. Although individual omics analysis, such as genome-wide association studies (GWAS), can effectively map variants correlated with AD, the majority of the heritability and the functional relevance of discovered variants are not explained or known by the identified variants. The limited success of singular approaches underscores the need for holistic and integrated approaches to investigate complex phenotypes using trans-omics data integration strategies. Integrating omics layers (e.g., genome, epigenome, transcriptome, proteome, metabolome, lipidome, exposome, microbiome), which often have complementary and synergistic effects, might provide the opportunity to capture the flow of information underlying AD disease manifestation. Overlapping genes/candidates derived from multiple omics types include *FLG, SPINK5, S100A8*, and *SERPINB3* in AD pathogenesis. Overlapping pathways include macrophage, endothelial cell and fibroblast activation pathways, in addition to well-known Th1/Th2 and NFkB activation pathways. Interestingly, there was more multi-omics overlap at the pathway level than gene level. Further analysis of multi-omics overlap at the tissue level showed that among 30 tissue types from the GTEx database, skin and esophagus were significantly enriched, indicating the biological interconnection between AD and food allergy. The present work explores multi-omics integration and provides new biological insights to better define the biological basis of AD etiology and confirm previously reported AD genes/pathways. In this context, we also discuss opportunities and challenges introduced by “big omics data” and their integration.

## Background

Atopic dermatitis (AD) is an inflammatory potentially debilitating skin disease associated with itch and eczematous lesions. It is primarily characterized by epidermal barrier dysfunction and immune alterations. Nearly 80% of children with AD progress to develop food allergy, asthma or rhinitis, underscoring its public health impact (1–3). Clinically, AD shows great patient-to-patient variability (probably representing multiple endophenotypes) and is associated with a wide range of abnormalities such as epidermal lipid and protease abnormalities, compromised cutaneous barrier function and inflammation. AD is diagnosed using patient history and visual assessment of the skin (as no specific laboratory test is available) and frequently managed with topical moisturizers, corticosteroids or calcineurin inhibitors—none of which are specific to AD (4, 5). A major gap that has hindered management strategies is the lack of treatment modalities tailored to the well-defined AD phenotypes that are still under investigation. It is widely accepted that integrative approaches linking multiple omics with clinical and epidemiological data are needed in order to develop better treatment and prediction models. Emerging biologic therapies, including cytokine-targeted therapies (e.g., anti–IL-4Rα mAb *Dupilimab* and anti–IL-31R mAb *Nemolizumab*), are costly but show promising results in patients sub-groups (6–8). Due to wide clinical variability, both topical and systemic therapies require better molecular tools/biomarkers for (a) identifying target patients to choose suitable treatment options and (b) assessing therapeutic outcomes.

**Figure 1:**
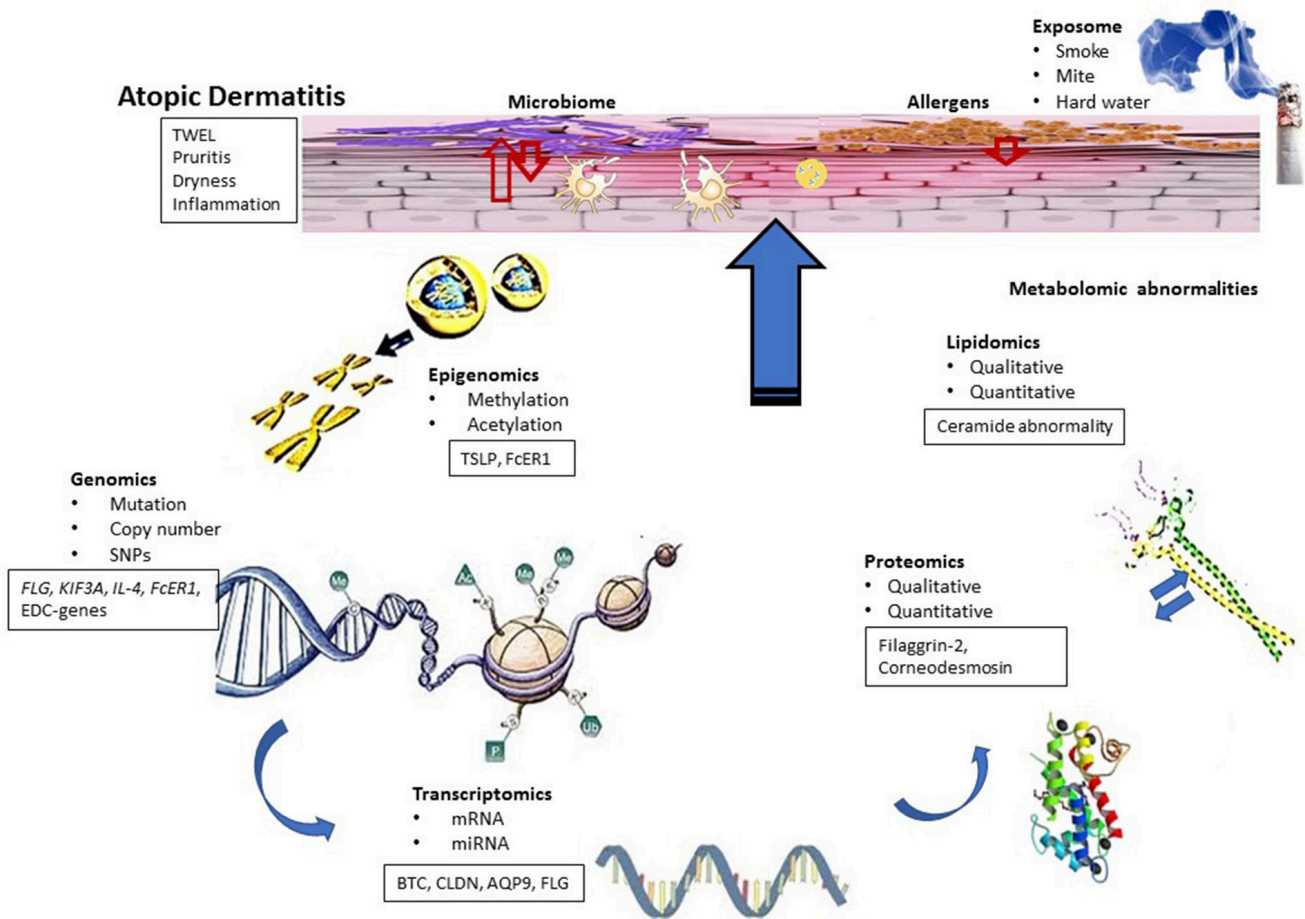
Conceptual biologic model for AD. Functional dysregulation in AD investigated using different omics techniques has been shown. AD skin is characterized by barrier dysfunction, inflammation and bacterial infection and associated with symptoms such as dry skin, itch, and inflammation. Genomic studies identified candidate genes that can be linked to transcriptomic studies. Some results also indicated the role of epigenetic modifications in AD, whereas transcriptomic studies identified functional clusters of genes related to AD pathogenesis. Differentially regulated genes of these functional clusters can be correlated with the results obtained from proteomic and lipidomic studies. On the other hand, differential regulation of innate immune genes and proteins can influence microbial diversity of the skin, which can also contribute to AD pathogenesis.

The promise of the multi-omics approach to decipher the endotypes of complex diseases has been well-described (9), and several investigators have proposed potential biomarkers and endotypes for AD using omics resources (10–12). However, although “omics-level” studies have been very useful in understanding the mechanism of AD manifestation (Figure 1), there have not been sufficient data nor attempts to integrate different omics data. This integration seems essential to interpret clinical variability and endotyping of AD. The present work outlines the roles of omics resources (such as genome, epigenome, transcriptome, proteome, metabolome, lipidome, exposome, microbiome) as parts of a puzzle and the approaches to integrate these layers and obtain an integrated systems-level overview of AD. Integrating multiple layers of omics information with clinical outcome data will be helpful in capturing the etiology of AD and its endotypes that can be useful in managing of this condition.

## The Pathobiology of AD is Incompletely Understood

AD has been renamed more than twenty times within the last 125 years, reflecting its wide clinical, genetic, and immunologic variability (11, 13). It is a disease of multifactorial etiology involving complex immunologic and inflammatory pathways (13, 14). The etiology of AD has been described using two opposing yet overlapping hypotheses. The “inside-out hypothesis” suggests that type 2 immune activation precedes cutaneous barrier impairment. Experimental data showing down-regulation of skin barrier genes by immune cytokines and mediators supports this hypothesis (15, 16). However, this hypothesis does not adequately explain the root cause of systemic inflammation, only highlighting the relevance of classical adaptive T_H_2 cells, in causing barrier dysfunction. In contrast, the “outside-in hypothesis” suggests that a broad skin barrier dysfunction, which precedes inflammation, is required for the manifestation of AD (16). This model is largely supported by data showing that the loss-of-function mutations in the filaggrin gene are linked to a subpopulation of AD patients (17). This outside-in hypothesis explains the causative role of epithelial barrier function in a subset of patients, as only about 20% of Northern European and Asian patients with AD bear known underlying defects in the barrier function, such as filaggrin mutations (compared to ~10% in non-AD population) (18). Moreover, in a study conducted by our group, ~39% of children with at least one parent with atopy developed AD by 3 years of age (19). This cannot be explained by *FLG* mutation alone, as only about 10% of AD cases could be projected from this mutation in general population (20). Thus, the “outside-in hypothesis” does not sufficiently explain the etiology of AD, showing the need to interrogate AD using novel approaches such as omics integration.

## Biological Variability and Endotypes—Challenges in the Management of AD

AD is clinically heterogeneous and lacks standardized laboratory assessment approaches. In the absence of a specific laboratory test to diagnose AD, evaluating the severity and treatment outcomes in AD patients are currently dependent on visually assessing the skin using clinical scoring such as EASI or SCORAD. However, these scoring systems are time-consuming and subject to human error (21). Recent efforts are also being made to incorporate cutaneous barrier function (determined by trans-epidermal water loss [TEWL]) and atopic status (cutaneous reactions to allergens, as measured by serum allergen-specific IgE with or without total IgE). The assessment is further complicated by factors such as (a) clinical variations, (b) age and ancestry/genetic variations of the subjects, and (c) experimental and omics-level variations. Heterogeneity in patients with AD concerning clinical features, age of onset, and genetic background has been studied by several groups (described below). Differences in sample collection (full thickness biopsy vs. skin stripping) and processing and tissue (blood vs. skin) used in the assays are known sources of data variability. Finally, investigators have used different “omics” tools (e.g., genomics, epigenomics, transcriptomics, proteomics) to study complex disease conditions like AD and generate potential omics candidates (e.g., candidate genes, transcripts, proteins, lipid mediators). However, thus far, there is little effort to either integrate the omics datasets or explore the overlap between omics candidates, which could potentially reduce variability.

The clinical variability in AD has been extensively reviewed by Bieber et al. (22). Interestingly, though both lesional and non-lesional AD demonstrate epidermal barrier defects as demonstrated by higher TEWL, significantly elevated allergen-specific serum IgE may (extrinsic AD; most frequent) or may not (intrinsic AD; also called non-atopic eczema) be present in symptomatic patients, indicative of two distinct subtypes, with higher IL-17 levels in intrinsic AD (23–25). Clinical phenotypic variability in AD may include time of disease onset (infancy, adolescence, adulthood), likelihood of persistence into adulthood, severity (mild-moderate, severe), co-existence of other allergic disease, co-existence of mendelian disorders associated with AD (e.g., Netherton's syndrome), IgE-mediated food or aeroallergen sensitization, presence of *Staphylococcus aureus* colonization and presence or absence of viral infections (e.g., eczema herpeticum) (22, 26).

In addition to clinical heterogeneity, the roles of age, gender, and race on manifestation/variability of AD symptoms have also been recognized. Esaki et al. demonstrated that young patients with AD (0–3 years old) may have a distinct immune activation pattern compared to that of older children with AD (27). Additionally, recent data indicated a relationship between a patient's ancestry and the underlying immune activation in AD. Noda et al. pointed out that Asian patients with extrinsic AD showed a phenotype with increased elongated epidermal rete ridges and parakeratosis similar to psoriasis (28). Skin biopsy specimens from Asian patients showed T_H_17/T_H_22-dominance compared to European American patients. The immune activation pattern in other ethnicities, such as African American, remains to be elucidated. Similar to clinical, age and race variability, treatment response variability is observed for AD. For example, promising yet variable response to treatments has been demonstrated in Dupilumab trials (reduction in Global Assessment score to 0 or 1 in 38% of patients) (7, 26, 29–31). Collectively, clinical, age, and race variability, along with differential response to treatment, in patients with AD demonstrated the need for patient-tailored treatment strategies (29).

Several endotypes of AD have been proposed on the basis of omics data (22). For example, Thijs et al. (12) analyzed 147 serum analytes (representing biomarkers for apoptosis, chemokines, growth factors, complement activation, epithelial cytokines, galectins, glucose regulation, immunomodulatory cytokines, inflammatory biomarkers, innate immunity biomarkers, leukocyte migration biomarkers, neutrophil/granulocyte biomarkers, proteases, and protease inhibitors, remodeling biomarkers, T_H_2 cytokines, vascular regulation, viral response and serum total and allergen-specific IgE) obtained from patients with moderate to severe AD (*n* = 200). A group of healthy, non-atopic subjects (*n* = 30) were used as controls. Principal component analysis revealed four clusters, each potentially representing a distinct endotype (12). However, neither candidate-focused nor global/omics-level information have been provided from other layers (e.g., epidermal differentiation complex gene variants, methylation, epidermal lipid composition, cutaneous *Staphylococcus* infection). Though serum-based studies provided useful hints about probable endotypes, interrogating more than one omic layers would be useful to better understand the root causes of clinical variability in AD.

Although a number of potential endotypes were suggested for AD on the basis of clinical phenotypes, the biomarkers for AD and AD endotypes are still under investigation. Biomarkers derived from an individual “omics” layer may not be sufficient to define all endotypes, underscoring the need to better connect candidate biomarkers to achieve an integrated systems-level view of AD pathogenesis and endotypes. Thus far, there are no specific treatments, nor biomarkers, particularly indicated for specific AD endotypes. Research is needed to connect AD endotypes suggested from studies involving individual “omics” layers.

## Unraveling the Pieces of the AD Puzzle: Role of Omics

Omics approaches have been applied to interrogate patient samples to identify endotypes and associated biomarker(s). Most studies, however, are restricted to only one omics layer, such as genomics or serum proteomics (12). Results interrogating multi-omics data, such as microbiome, proteome, lipidome, transcriptome, epigenome, exposome, and genome, are largely lacking. Publicly accessible databases can serve as powerful resources of omics-level data to unravel new biological insights into the etiology of AD and to confirm previously reported AD genes and pathways (32).

### Genomics Data

International collaborative efforts have generated genome informatic resources and databases, such as HapMap, 1000 genome and dbSNP, that are accessible to researchers searching for gene variants, mutations and other population genetics information. The genome-wide association study (GWAS) catalog (https://www.ebi.ac.uk/gwas/) represents a database of published GWAS. There are at least 12 GWAS for AD. Mining the GWAS catalog using “AD” as the query term showed genes (*FLG, KIF3A*) that are also known as AD-relevant genes from global expression studies (33–35). Interestingly, all GWAS indicated that AD is associated with immune regulation genes and cutaneous barrier function genes as indicated in Table 1 (43–47). In addition, a recent GWAS has indicated that asthma, hay fever and eczema partly coexist owing to their shared genetic risk variants leading to dysregulated expression of immune-related genes (48). AD-associated genes can be retrieved from the GWAS catalog and used for an enrichment analysis to identify significant disease-associated pathways. Molecular and cellular biologists may also query specific genetic variants for levels of gene expression in different tissues by searching GTEx and pathways from KEGG and Reactome (49–52).

**Table 1:** AD genome-wide association studies.

**Associated gene**	**Location**	**Function**	**References**
*C11orf30, LRRC32*	11q13.5	Immune regulatory	Esparza-Gordillo et al. (36)
*FLG*	1p21.3	Skin barrier	Sun et al. (37)
*SLC25A46, SLC7A9*	5q11.1	Solute carrier function
*TMEM232*	5q11.2	Integral membrane function
*TNFRSF6B, ZGPAT*	20q13.3	Immune response
*OVOL1*	11q13	Skin barrier	Paternoster et al. (34)
*ACTL9*	19p13.2	function not clearly defined
*KIF3A, IL4, IL13*	5q31	Intracellular transport, Cytokine
*IL1RL1, IL18R1*,	2q12	Immune response	Hirota et al. (38)
*CCR4, GLB1*	3p21.33	Immune response
*CCDC80*	3q13.2	Skin barrier
MHC region	6p21.3	Immune response
*CARD11*	7p22	Immune response
*ZNF365, EGR2*	10q21.2	Immune regulatory
*OR10A3, NLRP10*	11p15.4	Immune response
*CYP24A1, PFDN4*	20q13	Vitamin D pathway
*BAT1*	6p21.33	Solute binding, enzymatic	Weidinger et al. (39)
*CREBL1*	6p21.33	Protein binding, regulatory.
*TNXB*	6p21.33	Protein binding
*LCE3E*	1q21.3	Protein binding, structural	Paternoster et al. (40)
*MRPS21*	1q21.2	RNA-binding, Ribosomal
*IL7R*	5p13.2	Cytokine function
*IL2RA*	10p15.1	Cytokine function
*ZBTB10*	8q21.13	DNA binding, regulatory
*SFMBT1*	3p21.1	Histone binding, regulatory
*VAX2*	2p13.3	DNA binding
*PCDH9*	13q21.31	Intracellular contact	Kim et al. (41)
*NBAS*	2p24.3	Cellular transport
*THEMIS*	6q22.33	T-cell regulation, function
*SCAPER*	15q24.3	DNA-binding, CDK2 regulation
*GATA-3*	10p14	Transcription factor
*PRR5L*	11p12	Regulatory, mTORC2 associated	Schaarschmidt et al. (42)
*CLEC16A*	16p13.13	Regulator of mitophagy

*GWA STUDIES involving AD have identified immune regulation genes and cutaneous barrier function genes. Principal AD-relevant candidate genes identified from GWAS, their chromosomal location and functions have been mentioned*.

Apart from the well-known null mutation, a general down-regulation of filaggrin (FLG) expression has been reported in AD (53, 54). *FLG* is the gene most replicated in AD GWAS among multiple ethnic groups. It is located in the chromosome 1q21 region, which represents a gene cluster encoding proteins involved in the epidermal differentiation. Clinical data indicate that *FLG* loss-of-function mutations may represent a patient sub-population with severe, early onset, extrinsic AD that may persist into adulthood (55). On the other hand, KIF3A is a member of the kinesin superfamily of microtubule-associated motors and is important for transporting protein complexes within cilia (56). Down-regulation of this protein may be related to insufficient aeroallergen clearance (56). Recent association studies have linked KIF3A with AD (57, 58) and childhood asthma (59). Although single-nucleotide polymorphisms (SNPs) have been identified for AD from GWAS, the functional implications and mechanisms of the associated loci are largely unknown. Genomic variants alone are not able to explain the changes in disease risk along an individual's entire life span. DNA, RNA, protein, and their metabolites often have complementary roles to jointly perform a certain biological function.

### Epigenomics Data

Epigenetic data can lead to information regarding heritable changes (methylation, histone modification and non-coding RNA-mediated gene silencing etc.) expression without involving modifications in the DNA sequence. Potential roles of epigenetic modifications in allergic diseases has recently been highlighted (60). A limited number of studies at the epigenome scale have been performed on samples from patients with AD to identify epigenetic signatures related to this condition. Hypomethylation of the promoters of *TSLP* and *FCERG* (encodes FcεRγ, a high-affinity IgE receptor chain) are responsible for over-expression of these genes in AD (61). Rodriguez et al. described significant methylation differences for various CpG sites between lesional AD skin samples vs. healthy control skin. These methylation differences partially correlated with differences in transcript levels of epidermal differentiation and innate immune function genes (62). Transcriptome data from this study can be obtained from NCBI GEO (GSE 60709) and used to identify pathway-level changes between lesional and non-lesional AD associated with detected epigenomic alterations.

Overexpression of the high-affinity IgE receptor on monocytes and dendritic cells can contribute to AD manifestation (63). Monocytes from patients with AD showed global-, as well as locus-specific hypomethylation at the *FCERG* promoter, compared to monocytes from healthy control subjects. Interestingly, this hypermethylation is inversely correlated with *FCERG* expression. Ziyab et al. also reported the synergistic roles of *FLG* genetic variants and differential DNA methylation on the development of AD (64). In another study, no significant difference was observed in genome-wide DNA methylation levels of whole blood, T cell, and B cell samples obtained from AD cases and controls (62). However, DNA methylation differences were not correlated in blood and skin samples, indicating that blood may not be an ideal surrogate for skin, but show the potential involvement of epigenetic factors in AD (62). In addition, two preliminary studies regarding the role of *FLG* methylation on allergy and AD were performed using buccal cell DNA or whole blood DNA and yielded conflicting results, potentially indicating the roles of tissue-specific epigenomic changes (64, 65).

Epigenetic databases, tools and resources are growing rapidly due to active research in this area, including the NIH Roadmap Epigenomics Mapping Consortium and the preparation of the human epigenome atlas (http://www.genboree.org/site/). The NCBI Epigenomics database was built as a repository for whole epigenetic data sets and was later merged to Gene Expression Omnibus (GEO). DiseaseMeth (http://202.97.205.78/diseasemeth/index.html) is a web resource hosting human disease–related, curated methylome data of more than 175 datasets extracted from methylation arrays and sequencing results, as well as scattered, aberrant methylation information. However, there are limited data available on the AD-related epigenome.

### Transcriptomic Data

AD transcriptome has been studied in patient samples cross-sectionally using microarray-based or RNAseq-based methods (66–68). Publicly accessible databases, such as NCBI GEO and EBI (European Bioinformatics Institute) Array Express, can serve as important resources of transcriptome data (32). Multi-origin transcriptome data obtained from public databases have been previously analyzed to obtain valuable insight into the AD disease profile (69, 70). Using this approach, our group recently developed a panel of 89 genes that can be used as a transcriptomic signature for AD (69). These differentially regulated genes were categorized under the following functional classes: (a) barrier function–related genes, e.g., *LCE2B, LOR*; (b) dysregulated lipid genes, e.g., *FADS1, FABP7*; (c) chemokine/cytokine genes, e.g., *CCL17, CCL18*; (d) protease and protease-inhibitor genes, e.g., *KLK5, SERPINB3*; (e) genes related to anti-microbial function, e.g., *MSMB, LTF*; and (f) genes of diverse metabolic functions, e.g., *ARGAP18*. Interestingly, the transcriptional signature from this unique combination of 89 genes could discriminate AD from control skin biopsy samples with a very high degree of predictive accuracy. Another meta-analysis–based study of the AD transcriptome data identified the relevance of the atherosclerosis signaling (IL-37, selectin E) pathways combined with the wide-range lipid and Th2-gene abnormalities in AD (70).

### Proteomic Data

With the availability of quantitative proteomic data resources, peptide abundance, modifications, and interactions are increasingly used to analyze molecules secreted by a variety of immune cells. High-resolution protein separation and mass spectrometry–based applications have generated a large volume of proteomic data that are deposited in proteomic databases for reuse (71, 72). Quantitative proteomic diversity in human tissues and organs in disease and health has been extensively investigated resulting in comprehensive human proteome databases (73, 74). The draft map of the human tissue proteome has been detected from > 80 % of the annotated protein-coding genes (75). Though the Human Proteome Project (HPP) was initiated by the Human Proteome Organization (HUPO) to understand the entire human proteome at the cellular level, its biology and disease-oriented branch (named B/D-HPP) supports state-of-the-art assessments of human proteome in health and disease (76). The Plasma Proteome Database (PPD) was developed by the HUPO as a part of the HPP, which shows great promise in understanding the plasma proteome function in health and disease (e.g., grouping plasma proteins with cardiovascular functions and their roles in heart disease) (77–79). However, thus far, there is no similar initiative to explore the AD proteome. Searching other proteomic databases, such as PRIDE (proteomics identifications database) (80–82) and GPM (global proteome machine) (83, 84), which represent very rich resources of raw proteomic data, using the search term “atopic dermatitis,” did not retrieve relevant results (search performed on 1st September, 2017).

Proteomic data related to AD can also be obtained by literature search from Uniprot, which is a well-known database of protein sequences and their functional information, some of which is derived from DNA sequences. Querying the Uniprot database using the search terms “atopic dermatitis” AND “organism:Homo sapiens (Human) [9606]” retrieved 27 AD-related proteins (retrieved on 1st September 2017). Selected studies reporting proteomic data are listed in Table 2 (85–87). In contrast to transcriptomic studies, skin proteomic studies thus far used skin taping to obtain samples. However, this method only collects samples from the stratum corneum, the upper skin layer. Thus, results using skin taping samples may not be directly comparable to those using full-thickness punch biopsy skin samples. However, GWA studies strongly indicate the roles of barrier- and protease functions of the *stratum corneum* in the development of AD. Particularly, filaggrin (*FLG*) mutations, low profilaggrin/filaggrin monomer expression and skin protease dysregulation have been found in AD. Comparing three independent, stratum corneum proteomic datasets, we identified 20 proteins involved in AD pathogenesis. The proteins in this set of 20 differentially regulated proteins were unique to certain datasets or represented in multiple datasets.

**Table 2:** Proteomics studies using skin samples obtained from patients with AD.

**References**	**Sample**	**Sample collection method**	**Detection method**	**AD-specific proteins identified**
**(A) PROTEOME-WIDE**				
Broccardo et al. (85)	Stratum corneum	Skin stripping	Mass spectrometry
Broccardo et al. (86)	Stratum corneum	Skin stripping	Mass spectrometry	153 proteins
Sakabe et al. (87)	Stratum corneum	Skin stripping Up to 20 layers	Mass spectrometry	421 proteins
**(B) PROTEIN-FOCUSED (TO VALIDATE GENE EXPRESSION DATA AT THE PROTEIN LEVEL)**				
Saaf (88)	Full thickness skin	Punch biopsy	Immuno-histochemistry	Socs3, TGase1, TGM1
Zeeuwen (89)	Cultured keratinocytes	Punch biopsy	Immuno-histochemistry	DEFB4, PI3
Gittler et al. (90)	Full thickness skin	Punch biopsy	Immuno-histochemistry	S100A7, S100A8
Suarez-Farinas et al. (91)	Full thickness skin	Punch biopsy	Immuno-histochemistry	LOR, FLG, CDSN

*Investigators used biopsy or skin taping (stratum corneum) to obtain samples. Proteomics studies were performed to check expression at either the global or candidate protein level (candidate proteins previously identified from genomics or transcriptomic studies). Sample types, methods and results have been summarized from independent studies*.

### Metabolomic Data

Metabolomic approaches use mass spectrometry and high-resolution proton nuclear magnetic resonance (NMR) to assesses cellular metabolites of multiple small molecule types (e.g., <1,200 Da and biochemical intermediates; i.e., metabolites), including amino acids, fatty acids, carbohydrates or other products of cellular metabolic functions, in order to explore disease metabolome related to allergic diseases (92–94). Information obtained from metabolomics provides useful insights about the biochemical phenotype (i.e., the metabotype). A limited number of metabolomic studies has been performed to analyze the AD metabolome using urine, serum and sweat analyzed by NMR and liquid chromatography–coupled mass spectrometry. In an exploratory study using NMR-based spectral analysis followed by statistical and chemometric approaches, Assfalg et al. noted significant spectral shifts indicative of broad changes in the AD urine metabolome compared to that of age-matched, non-atopic controls (95). Subsequently, Huang et al. used high-performance liquid chromatography–coupled mass spectrometry to investigate metabolic abnormalities in AD using fasting sera obtained from children with AD and healthy control subjects. Subjects with AD with high IgE showed significant differences in multiple metabolic intermediates, including carnitines, free fatty acids and lactic acids, indicating metabolic abnormalities. In contrast, higher levels of cytochrome P450 and epoxygenase metabolites were reported in the AD group with normal IgE. Statistical modeling discriminated high IgE AD from low/normal IgE AD and control samples in scattered plots. In a targeted metabolome analysis, the investigators analyzed thirty eicosanoids, which are products of the arachidonic acid pathway and mediate inflammation.

There are several recently released metabolomic databases in use. Examples include HMDB (The Human Metabolome Database), BiGG, and SetupX, among others (96–98) The HMDB is a publicly accessible database that contains detailed information regarding small molecule metabolites related to the human body (96, 99, 100). This database is designed for applications in metabolomics, clinical chemistry, and biomarker discovery. However, none of these databases presently contain AD-focused or atopy-related metabolome data.

### Lipidomic Data

Lipidomics involves system-wide identification and quantitation of lipids with lipid-associated pathways and networks. Lipids play distinct roles in mediating inflammation, as well as maintaining the skin moisture content. Genomic, transcriptomic and proteomic data strongly indicated the involvement of cutaneous lipid dysregulation in AD. However, only a few studies have directly addressed AD lipidomics thus far. A recent study aimed to characterize the lipid mediator profile in sweat samples obtained from patients with non-lesional AD and non-atopic controls (*n* = 23/group) using liquid chromatography–mass spectroscopy and mass spectroscopy. Increases in the C30–C40 ceramide and C18:1 sphingosine concentrations were found in AD patients despite no differences in TEWL between study groups, and this effect was strongest in men. No differences in oxylipins and endocannabinoids were observed between study groups. The increases in short-chain lipids do not support the current report indicating a general ceramide deficiency in AD (101). However, an imbalance between different ceramide groups was observed, indicating that ceramide species involved in barrier function and keratinocyte signaling are dysregulated in AD. Lipid abnormalities in AD have also been shown using skin and sweat samples (102–104).

Searchable interactive databases for lipids and lipid-associated proteins, in combination with mass-spectrometric and other experimental approaches, offers an opportunity to construct lipid metabolic networks and to devise therapeutic strategies (105–108). LMSD (lipid maps structural database) contains structures and annotations of biologically relevant lipids. Lipidomic pathways and networks can be constructed from experimental data. Interrogating the epidermal lipidome has been described as a tool to assess and predict the progression of inflammatory skin diseases (109). In spite of the recent reports showing the roles of epidermal and sweat lipidome in AD, relevant lipidomic data generated by independent investigators are not usually made available through publicly accessible databases (104, 110–112).

### Microbiome Data

The human skin is colonized by microorganisms, including bacteria, viruses and fungi, collectively known as the microbiota, and their genes constitute the microbiome. Skin microbiomes in AD have been studied in the recent years using samples obtained during in-flare and out-of-flare conditions (113, 114). AD treatments can also lead to increased epidermal bacterial diversity preceding improvements in symptoms. *Staphylococcus* sequences (mostly *S. aureus*) were more abundant during disease flares (lower microbial diversity), compared to baseline or post-treatment conditions (higher microbial diversity), and correlated with disease severity (113, 115). Modern high throughput studies also indicated a higher representation of skin commensal *S. epidermidis* during flares which was not previously demonstrated in culture-based studies (113). Increases in *Streptococcus, Propionibacterium*, and *Corynebacterium* species were observed following therapy. These findings reveal connections between microbial communities and inflammatory diseases such as AD and demonstrate that high resolution examination of microbiota associated with human disease can provide novel insights into global shifts of bacteria relevant to disease progression and treatment.

Interestingly, altered epidermal lipid composition has been found to correlate with the status of *S. aureus* colonization on the AD skin (116). In general, a number of studies indicated a clear difference between AD- and healthy skin microbiome, which can be associated with disease state, i.e., appearance of flares (113, 117, 118). AD skin often shows *S. aureus* infection, which exacerbates the disease through inflammatory mechanisms. Microbiome studies indicated that coagulase-negative *Staphylococcus* strains having antimicrobial activity are common in the normal population but rare in subjects with AD. The antimicrobial activity has been related to previously unknown antimicrobial peptides produced by coagulase-negative species such as *Staphylococcus epidermidis* and *Staphylococcus hominis*. Our present knowledge clearly indicates that some commensal bacteria, that are deficient in AD skin, can protect against harmful *S. aureus* infection and can have a great translational value in reducing infection and inflammation (119). Another study showed that topical corticosteroids might influence the lesional AD microbiome, with the post-treatment AD skin microbiome resembling the non-lesional AD skin microbiome but both exhibiting distinct characteristics from the normal skin microbiome (120). Additional studies on gut microbiome have also suggested that alterations in the gut microbiome may be associated with an altered host immune function related to AD.

Over the last few years, identifying the composition of microbiome has progressed enormously with the application of high-throughput DNA sequencing technologies based on two principal methods. The first method is based on sequencing the 16S rRNA gene of bacteria (121). Identifying variations in specific regions of this gene allows decent classification of bacterial taxa. 16S is relatively inexpensive, but has limited resolution (not all bacteria can be classified, species-level identification may not be possible), nor can the 16S method detect viruses and eukaryotic communities. The second method, metagenomics sequencing, is based on sequencing of all DNA fragments and aligning them to reference databases from all life forms (122). This approach is capable of identifying bacterial, viral, fungal, and protozoan DNA. It produces a much better resolution of bacteria at the species level. However, this method is associated with high sequencing costs, significant bioinformatic load (due to the large number of sequence reads produced), and the inability to analyze genomes not present in the reference databases or genes with unknown function. The statistical methods widely used in microbiome analysis mostly derived from ecological studies, including alpha diversity (diversity of species or other taxa within a sample) and beta diversity (difference in taxonomic composition between samples). However, unlike ecological studies, microbiome researchers are dealing with relatively large numbers and employ additional multidimensional statistics and complex statistical approaches, including pathway and network analysis. The NIH human microbiome project (HMP) has generated data to interpret the role of the microbiome in human health and disease. This project has generated a large volume of publicly accessible metagenomic sequence data from five major body locations (including skin and gut) of healthy and disease (preterm birth, inflammatory bowel disease, and type-2 diabetes) cohorts. More AD-focused data is required.

### Exposome Data

The exposome is defined as the totality of environmental exposures over the life course, with exposure timings ranging from prenatal to post-natal periods. It is now well-recognized that human omics alone cannot explain the rise in allergic diseases in the industrialized world, clearly indicating the role of the exposome in allergic diseases including AD (123). Birth cohort studies indicated that prenatal exposure to antibiotics through expecting mothers' antibiotic consumption might be associated with an increased risk for AD, particularly within the children of mothers with atopy. Antibiotic exposure also might occur through breast feeding. Both prenatal and post-natal exposure to antibiotics may influence intestinal microbial diversity of the neonates, leading to subsequent manifestation of atopic diseases (124–126). Other prenatal exposures include oxidative stress induced by maternal distress (127). maternal exposure to air pollutants (128, 129), smoke (130, 131), phthalate (132), cadmium (133), house dust mite (134), house pets, and farm animal (protective effects) (135).

Numerous studies have been designed to assess the role of post-natal exposures on AD. There is growing evidence that the use of hard water might be a risk factor for developing AD (136–138). According to a recent study, fall/winter birth season and exposure to hard water were associated with increased relative prevalence of AD in the first 18 months of life (136). Other potential factors included meteorological factors (139) and pollutants (139–141), allergenic grass pollen (142) mold (143, 144), probiotics (145–147), and vitamin D exposure (148–150). Although, high-throughput techniques have been developed and have been widely used for omics such as transcriptomics, proteomics, metabolomics; exposure to environmental stressors affects each of these components (genes, transcript, proteins, metabolites). Thus, there is a need to combine omics with environmental exposure measures to better understand the complex network of cellular responses to the exposome.

Exposome-Explorer (http://exposome-explorer.iarc.fr) is a database supported by the World Health Organization. It is dedicated to biomarkers of exposure to environmental risk factors (151). It contains detailed information on the nature of biomarkers (e.g., dietary and pollutant) and their concentrations in various human samples (e.g., blood, urine). Recently there is a great interest in exposome studies to identify disease risks. However, thus far, there are limited data pertinent to AD in exposome databases.

## Omics-Wide Integration—Putting the Pieces Together

Each omics data type typically provides a list of differential factors potentially associated with the disease. These data can be useful as disease markers while providing insight as to which biological pathways or processes are different between the disease and control groups. However, analysis of only one omic(s) data type is limited to correlations and provides a partial view of the biological system. Integrating different omics data types is often used to elucidate the potential causative changes that lead to disease or can be used to identify potential therapeutic targets for further molecular studies. For example, a GWA study can reveal the statistical associations between genetic loci and disease status. While it can suggest potential causal effects, pinpointing particular causal variant(s) and associated molecular mechanisms can be challenging. On the other hand, gene expression or epigenomic profiling can detect associations between disease and genes or epigenomic markers, but these associations are correlative in nature. By integrating different types of data, it becomes possible to circumvent the limitations of individual studies and better identify disease-causing DNA variants and their downstream molecular targets. For instance, when DNA and RNA are measured simultaneously, it is possible to determine whether a particular genetic variant affects the downstream expression of a gene in a “genetics of gene expression” (expression quantitative trait loci; eQTL) analysis. Furthermore, if a genetic variant resides in a functional site associated with transcription factor binding, epigenetic modification, or protein regulation, as revealed by the ENCODE project, it becomes possible to narrow the basis of potential targets. Co-analyses of genomic data with expression profiles from either the transcriptome, proteome or methylome help to identify the quantitative trait locus and eQTL, proteome QTL, or methylome QTL. Integrating transcriptome and proteome data has also led to the discovery of post-translational activities and correlation between two omics types under identical conditions. A network-based method, along with omics data integration approaches, will be discussed in the data integration section.

### Omics Integration to Address AD Etiology and Clinical Variability/Endotypes

Clinical management of AD is often challenging due to its heterogeneity with multiple endotypes (e.g., intrinsic vs. extrinsic, with or without other allergic or immune diseases, mild or moderate vs. severe forms, *Staphylococcus*-infected vs. uninfected forms). Therefore, much of current research has been focused on gaining a better understanding of the endotypes with omics data. However, these studies are often underpowered. In an attempt to connect endotypes with transcriptome data, Martel et al. (152) described that in mild, extrinsic AD (total IgE >200 kU/L; *n* = 5), the expression of most skin barrier genes, including *filaggrin* and *loricrin*, remained unchanged (or moderately upregulated) compared to controls (*n* = 9), despite the presence of heterozygous mutations in the *filaggrin* (*FLG*) gene in majority of patients. On the other hand, mild, intrinsic AD (*n* = 9) resembled mild psoriasis (*n* = 9), rather than AD, when expression profiles of genes involved in inflammatory responses were compared between these conditions (152).

In AD, the skin is infected with viral and bacterial strains in at least 30% of patients, while about 90% of active skin lesions are associated with *Staphylococcus* infection (153). However, studies so far have not been performed to directly connect the skin microbiome with transcriptome or any other omics layer in AD. Transcriptome studies have linked infection with down-regulation of innate immune genes (such as genes coding antimicrobial peptides) in the epidermis (69). Down-regulation of a wide array of innate immune genes, like *LTF, MSMB, RNASE7*, and *SCGB2A1*, has also been shown from AD transcriptomic studies, which might be linked to increased susceptibility to microbial infection in AD (69).

Filaggrin is important for the formation of *stratum corneum* layer and also for its hydration of this barrier properties. Subjects carrying mutations in the *FLG* gene produce dry, flaky skin and potentially get sensitized to allergens due to enhanced exposure (154). To assess the effect *FLG* loss-of-function mutations on skin transcriptome, uninvolved skin of pediatric AD patients (*n* = 26) has been compared with site-matched samples from controls (*n* = 10 non-atopic) (155). Cases and controls were screened for *FLG* genotype to stratify the transcriptome data. Interestingly, *FLG* wild-type cases showed dysregulation of genes involved with lipid metabolism, while *FLG* haplo-insufficiency affected global gene expression and was characterized by a type 1 interferon–mediated stress response. This study, along with clinical stratification based on *FLG* status, suggested that subjects with the *FLG* loss-of-function mutation might represent a specific endotype characterized by early onset disease with more severe and generalized manifestation of symptoms (156) However, there is an insufficient number of genomics studies to further define AD endotypes.

### Omics Integration: Biological Relevance and Approaches

Genes identified from genomic (GWAS), epigenomic and proteomic studies can be used to build AD disease- associated networks from each omics level and a network-level overlap can be calculated. Previous studies identified centrally located network genes or “hub genes” from individual omics layers (69). For a more integrated systems level overview, AD-relevant candidates (genes/transcripts/proteins) from each omics level could be combined to build a disease network. Significant AD-relevant genes can be prioritized from omics databases, or from experimentally derived multi-omics data available from existing literature using standard literature interrogation tools (157). This comprehensive approach (inclusive method) uses advanced analytics to explore causal relationships between omics candidates and disease conditions/ pathways giving insights not achievable from any single-layer omics data.

**Figure 2:**
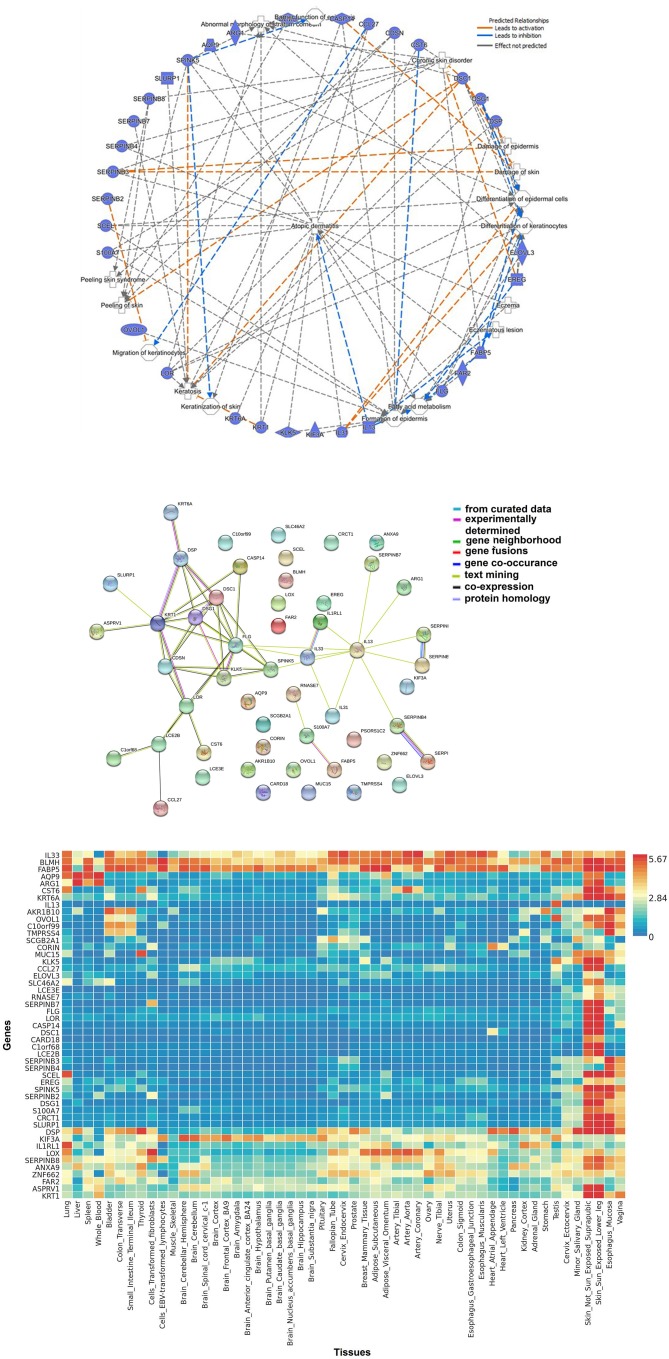
Biological approaches of omics integration: **(A)** Network of significant AD-relevant candidates from multi-omics data. These candidates contain members from different functional groups such as keratinocyte development, cutaneous barrier function, inflammation, fatty acid metabolism, and cytokine function. **(B)** Protein-protein interaction networks: functional interaction was visualized using STRING protein network. STRING (http://string-db.org/) is a database of known and predicted protein interaction, including physical and function association. STRING quantitatively integrates interaction data derived from genomic context, high-throughput experiments, co-expression and previous knowledge [Szklarczyk et al. (158)]. Connections, based on co-expression and experimental evidence have been shown. Filled nodes indicate genes; edges between nodes show protein-protein interactions between protein products of the corresponding genes. Different edge colors represent the types of evidence for the connection. **(C)** Tissue-based expression heatmap to directly explore the functional relevance of AD-relevant candidates/genes clustered by genes and by tissue type. The heatmap indicates the relatedness of skin and esophagus in the context of AD-relevant candidates.

Figure 2A represents network of 52 AD-relevant candidates from multi-omics approach (Ingenuity Pathway Analysis, Qiagen, USA). These candidates contain members from different functional groups such as keratinocyte development, cutaneous barrier function, inflammation, fatty acid metabolism and cytokine function. Their functional interaction can be visualized by STRING protein network (Figure 2B). STRING v10.0 (http://string-db.org/) is a database of known and predicted protein interaction, including physical and function association (158). Furthermore, these candidates can be used to develop a tissue-based expression heatmaps to directly explore their functional relevance in disease-relevant target tissue(s). Figure 2C represents a tissue-resolved expression heatmap of 52 AD-relevant candidates using FUMA (a functional genomic annotation tool) (159). The heatmap, when clustered by AD genes/ candidates and by target tissues, shows the relatedness of skin and esophagus in the context of AD-relevant candidates, which potentially explains the shared etiology between AD and food allergy. Our findings suggest that the pathways shared between AD and other allergic diseases might have significant functions in skin, esophagus, vagina—areas characterized by shared structural protein components. Particularly, the functional significance of structural proteins co-expressed by oral and skin tissues have been described (160). By applying gene ontology (GO) enrichment analysis, we found that these candidate genes are associated with epidermal development, keratinocyte differentiation, and epithelial cell differentiation pathways were most differentially enriched in asthmatics. Interestingly, many of these pathways derived from these candidate genes have also been related to Th2 pathway, immune cells, and response to cytokine.

**Figure 3:**
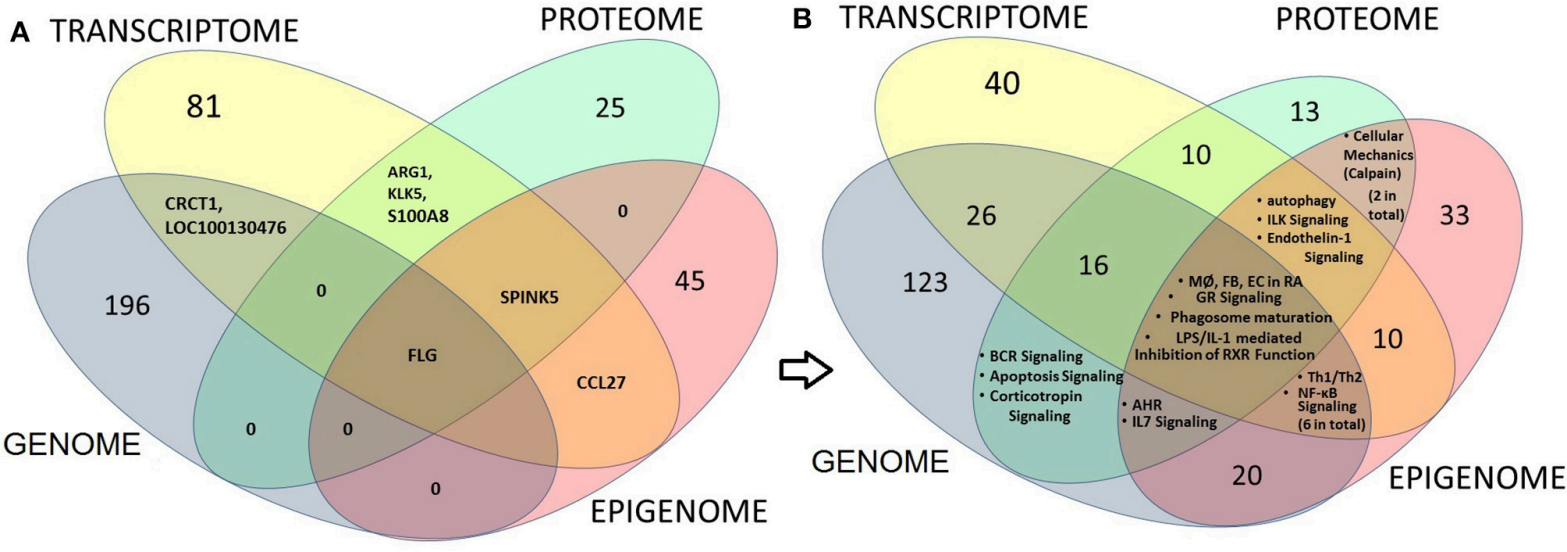
Multi-omics overlap analysis at gene and pathway level to integrate and identify AD candidate loci (at gene, transcript or protein) level (left panel) or at the pathway level (right panel). The genomic, epigenomic, transcriptomic and proteomic candidates that are associated with AD have been obtained from GWAS, from published epigenomic, Transcriptomics, and proteomic data respectively. Venn diagrams show the overlap between candidate gene, transcript or proteins (left panel; **A**) or between pathways (right panel; **B**) derived from each level. Numbers indicate the numbers of shared candidates or pathways. The result shows that only one candidate i.e., filaggrin (FLG) could be associated with AD from four omics (Genomic, epigenomic, Transcriptomic and proteomic) level experiments, followed by SERPINB3 (reported from most transcriptomic, epigenomic and proteomic studies; **A**). Interestingly we noticed that although there is a limited overlap between candidates obtained from individual omics-level data, while the candidates demonstrated considerable overlap at the canonical pathway levels showing the significance of macrophage (MØ), endothelial cell (EC) and fibroblast (FB) activation pathways (from GWAS, transcriptome and Epigenome—four omics levels) as found in rheumatoid arthritis (RA), as well as Th1/Th2 activation and NFkB activation pathways (GWAS, transcriptome and Epigenome) and autophagy, ILK and Endothelin-1 signaling pathways (transcriptome, proteome, and epigenome) in AD **(B)**.

We further ran an overlapping analysis of candidate genes identified from multiple omics levels. Figure 3 represents an omics data integration by multi-omics overlap. The genomic, epigenomic, transcriptomic and proteomic candidates that are associated with AD have been obtained from GWAS catalog and from published epigenomic, transcriptomics, and proteomic experiments. Individual omics datasets were analyzed, and Venn diagrams were produced to visualize the overlap between genes and pathways. The result shows that filaggrin was associated with AD from four omics (genomic, epigenomic, transcriptomic, and proteomic) level experiments, followed by SERPINB3 (reported from most transcriptomic, epigenomic, and proteomic studies). The number of candidates from each omic level might increase, as more data will be available from different levels. Interestingly although we observed a limited gene level overlap between different omics-level data in AD (candidate included SPINK5, KLK5, FLG, CRCT1, ARG1, S100A8, and CCL27 reported ≥2 omics level overlap) there was a remarkable overlap at the pathway level. The pathway-level overlap included macrophage, endothelial cell and fibroblast activation pathways, Th1/Th2 lymphocytes and NFkB activation pathways.

The potential role of NFkB in the manifestation of inflammatory responses in AD is well-described (161). In fact, NFkB -targeted therapies, although not very specific to AD, have shown promising results in a mouse model of AD (162–164). The role of Th1/Th2 lymphocytes in AD has been the subject of many studies, but the significance of other cells and tissues, particularly macrophage, endothelial cell and fibroblasts, is relatively unexplored. However, their involvement in AD is strongly indicated by our multi-omics approach. Experimental data indicated that fibroblasts from the skin biopsy samples of patients with atopy modulate the proliferation and terminal differentiation of keratinocytes obtained from control subjects; this modulation leads to alteration of the keratinocytes' morphologic features, which could be restored by healthy fibroblasts (165). These effects were linked to low expression of the differentiation-associated cytokine leukemia inhibitory factor by fibroblasts from subjects with AD (165). On the other hand, experimental data also demonstrated that granulocyte macrophage colony-stimulating factor (GM-CSF) was overproduced by keratinocytes in AD, which was linked to sustained dendritic cell activation in AD skin (166). A significant role of macrophages in the manifestation of AD has been reviewed by Kasraie et al. (167). Experimental data further demonstrated an increased macrophage infiltration of the skin and interactions through surface-bound IgE and by secreting chemokine mediators. Human macrophages are also activated through its surface-expressed IL-31RA, particularly when exposed to staphylococcal enterotoxin in AD (168). Finally, the central role of epithelium has long been recognized in the pathogenesis of asthma and AD (169). Skin and airway epithelium produce TSLP, which directs the cross-talk of epithelium and dendritic cells to augment allergic inflammation. It can activate neurons to induce itch in AD (170). Epithelium-derived TSLP can also directly initiate Th2 type cytokine production by mast cells, providing a T cell–independent route to pro-allergic responses (169). Interestingly, since TSLP induction occurs through epithelial Toll-like receptors, TSLP-mediated allergic manifestations represent an important link between innate immunity and allergic disease (169). In summary, published experimental data strongly support our multi-omics results, which demonstrate the role of other cells (i.e., macrophage, endothelial cell, fibroblasts) beyond the lymphocytes and of barrier function proteins (e.g., filaggrin, occludin, claudin) in AD.

Our multi-omics approach also suggests the roles of autophagy, integrin-linked kinase (ILK) signaling and endothelin 1 (ET-1) signaling in AD. Though ILK signaling has not been well-explored in the context of AD, there are reports showing that autophagy and ET-1 might play very significant roles. ILK activation might be a result of *S. aureus* infection, which is frequently associated with AD skin (171). The role of autophagy in regulating keratinocyte inflammation in case of skin diseases, including AD, has been previously discussed (172–175). Interestingly, experimental data also suggest that *Staphylococcus* strains might persist within keratinocytes by stimulating autophagy (176). This promotes the degradation of inflammasome components and facilitates *Staphylococcal* survival. Thus, autophagy is relevant in explaining the persistent, methysilate-resistant *Staphylococcal* infection in AD (176). However, the roles of ET-1 in AD have been better explored. ET-1 functions as a pruritogenic mediator in the manifestation of AD symptoms (177). Its multifaceted roles in AD have been investigated by several groups, and its plasma concentration has been correlated with AD disease severity (178–180).

Biological data from multi-dimensional information levels are highly inter-connected and when combined, can provide meaningful insights. Genetic variants are subject to epigenetic regulation, which helps determine the level of gene expression producing “intermediate phenotypes” and ultimately alter an individual's risk of disease development or disease severity. For example, a combined epigenetic-transcriptomic analyses of epidermal samples obtained from AD lesions compared to healthy epidermis, indicated differences in methylation status, which partially correlated with differentially regulated transcript levels of epidermal differentiation and immune response genes (61, 62). Although there is a limited number of reports in the literature that use a multi-omics approach to interrogate AD samples, additional results from multi-omics studies are expected in the future due to the increasing availability of cost-effective omics tools that can generate big data utilizing a minute amount of a patient sample. However, this requires collecting samples for each subject for multiple omics applications. Ongoing research is directed toward utilizing minimally invasive methods, such as tape striping, which could help obtain samples (microbiome, DNA, RNA) from the same affected site for multi-omics experiments.

## Approaches to Integrate Omics-Wide Data

Omics resources and omics integration tools exist (represented in Table 3). The rationale to develop systems biology technologies that integrate multiple omics data types for molecular taxonomy of complex diseases has been emphasized by several authors (9). However, this approach has not been used in AD so far. Computational biology tools have emerged in the recent years for integrating high-throughput data using multistage and meta-dimensional approaches to meet the demands for within and between omic-level integrations. An integrative approach to interpret multi-omics data can significantly alleviate the bottlenecks by providing valuable insights may not be achievable by any single-layer omic approach. Gene network and pathway modeling approaches to integrate resources from different omics domains are being developed to address this knowledge gap (9, 181–187). Network analysis investigates the functional and physical interaction among genes (188). A widely used approach to translate diverse analytic data into biological understanding is by projecting and visualizing the data onto curated biological pathways (e.g., KEGG). Bioinformatics and visualization tools allow the investigators to identify co-regulated genes, metabolites, and proteins in an intuitive and easy-to-use manner. For example, pathway-based analysis tools that integrate GWAS with curated pathways and accept eQTL information have been developed (189, 190). In addition to knowledge-based pathway analysis, data-driven approaches that utilize gene regulation and protein-protein interaction networks could be applied to identify the most likely pathologic perturbations and target genes for disease-associated loci.

**Table 3:** Publicly available omics resources relevant to AD.

**Omics type**	**Database name**	**Web link**
Genomics	HapMap	http://hapmap.ncbi.nlm.nih.gov/
	1000 Genomes Project	http://www.1000genomes.org
	GWAS Catalog	www.genome.gov/gwastudies
Epigenomics	HEA	http://www.genboree.org/
	NCBI Epigenomics	http://202.97.205.78/diseasemeth/index.html
Transcriptomics	Gene Expression Omnibus	https://www.ncbi.nlm.nih.gov/gds/
	ArrayExpress	https://www.ebi.ac.uk/arrayexpress/
Proteomics	Plasma Proteome Database	http://www.plasmaproteomedatabase.org/
	PRIDE (proteome identification)	https://www.ebi.ac.uk/pride/archive/
	Expasy	https://www.expasy.org/
Metabolomics	HMDB	http://www.hmdb.ca/
	BiGG	http://bigg.ucsd.edu/
Lipidomics	LMSD	http://www.lipidmaps.org/data/structure/
	Lipidblast	http://fiehnlab.ucdavis.edu/projects/lipidblast
Microbiome	Human Microbiome Project	https://hmpdacc.org/hmp/
Exposome	Exposome-Explorer	http://exposome-explorer.iarc.fr
Integrative omics	IMPALA	http://impala.molgen.mpg.de/
	iPEAP	http://www.tongji.edu.cn/?qiliu/ipeap.html
	MetaboAnalyst	http://www.metaboanalyst.ca/
	SAMNetWeb	http://fraenkel-nsf.csbi.mit.edu/samnetweb/
	pwOmics	https://bioconductor.org/packages/release/bioc/html/pwOmics.html
	WGCNA	https://horvath.genetics.ucla.edu

*Biological databases containing omics data and their web-links have been shown. Representative omics integration tools based on biochemical pathways and ontology, network-based and empirical correlation–based methods have been mentioned. HEA, Human Epigenome Atlas; PRIDE, Proteomics data Repository; HMBD, Human Metabolome Database; BiGG, UCSD Big data modeling resources; LMSD, LIPID MAPS Structure Database; IMPALA, Integrated Molecular Pathway Level Analysis; iPEAPS, integrating multiple omics and genetic data for pathway enrichment analysis; pWOmics, pathway-based level-specific data comparison of matching omics data sets; WGCNA, Weighted correlation network analysis, also known as weighted gene co-expression network analysis. Weblinks to respective resources have been provided*.

Three data integration techniques are widely used: (a) biochemical pathway- and ontology-based, (b) network-based and (c) empirical correlation-based methods (191). (a) Biochemical pathway- and ontology-based methods are based on enrichment analysis in individual omics levels and thus are highly sensitive to the expert definitions of the constituent pathway members. In the most complex disease types, candidate genes and proteins can be integrated at the pathway level rather than at the candidate gene and protein levels. (b) Network-based approaches utilize biological networks involving individual genes, proteins and metabolites that can be used to generate an integrated map of multiple omics-level with results independent of any predefined biochemical pathways. (c) Empirical correlation-based approaches are useful for biological and other meta-data (e.g., clinical outcomes) integration, particularly in the absence of other domain knowledge.

A large number of publicly available tools have been developed for omics data integration based on the above-mentioned approaches (191). Integrated molecular pathway level analysis (IMPALA) is a widely used online tool that accepts gene or protein expression and metabolomics data as inputs and identifies novel pathways from the combined datasets on the basis of over-representation and enrichment analysis from a large number of pre-annotated pathways linked to multiple databases (192, 193). A similar approach has been used by other programs such as iPEAP and MetaboAnalyst (194, 195). iPEAP accepts transcriptomics, proteomics, metabolomics, and GWAS data, whereas MetaboAnalyst accepts data from either targeted profiling (concentration tables) or metabolic fingerprinting approaches (spectral bins, peak lists) from NMR, LC-MS, or GC-MS and performs a huge number of statistical analyses for pathway and biomarker analyses that can be correlated with other omics layers (195, 196). Additional tools are continuously developed to integrate metabolomic data with genomic and pathway data (e.g., RaMP) and transcriptomic data (197, 198). Network-based program suites are available for either online use or installing locally as “R” packages/cytoscape plug-ins (199–201). For example, SAMNetWeb is a web tool capable of accepting two distinct data types (e.g., transcriptome and proteome data) across multiple experiments to identify and visualize activated pathways (199). On the other hand, programs such as pwOmics and metamapper are available as “R” packages (191, 202).

Several empirical correlation-based methods are developed to integrate multi-omics data. For example, weighted correlation network analysis (WGCNA) is an “R” package that can be used for finding clusters of highly correlated genes (gene clusters) (203). It can also be used to connect gene clusters to additional information, such as single-nucleotide polymorphisms (SNPs) and proteomic and clinical data, and thus can be used to identify candidate biomarkers or therapeutic targets (191, 203). These methods have been widely applied in various biological/ biomedical contexts (e.g., cancer, mouse genetics, yeast genetics, brain imaging data) (191). Several powerful omics integration packages have been developed for additional data integration including physiological conditions, biochemical reactions, molecular and mass-spectral similarity. Interestingly, the Grinn package provides a dynamic interface to rapidly integrate gene, protein and metabolite data using both biological (network–based) and empirical (correlation–based) approaches (http://kwanjeeraw.github.io/grinn/).

In addition to omics data, there are novel health data resources that are expected to revolutionize personalized medicine. For example, big data analytics in personalized and predictive medicine of complex diseases, including allergic disorders, has been previously emphasized by several authors (204–206). Leveraging big data (using emerging, data-science tools such as deep-learning) can be very efficient in obtaining insight into complex disease outcomes and suggesting the best use of healthcare resources. Beyond traditional data sources (e.g., electronic health record), novel tools capable of recording personal health profiles captured by individuals themselves are going to contribute a high volume of data to the systems biology of complex diseases. Though traditional omics data represent snapshots from individuals at research and clinic visits, big data can capture intrinsic, and extrinsic variables in real time through recording using novel technologies such as wearable devices and mobile health apps (205). Patients and control subjects can record parameters related to skin care, food, exercise, adherence to medication, and others. In addition, modern sensor technologies allow recording of several parameters, such as physical activity, blood pressure, glucose level and heart rate, but can be extended to include measures of TEWL, lung function, symptom scores, humidity and environmental exposures (205, 207).

## Approaches to Connect Omics to Endotypes

**Figure 4:**
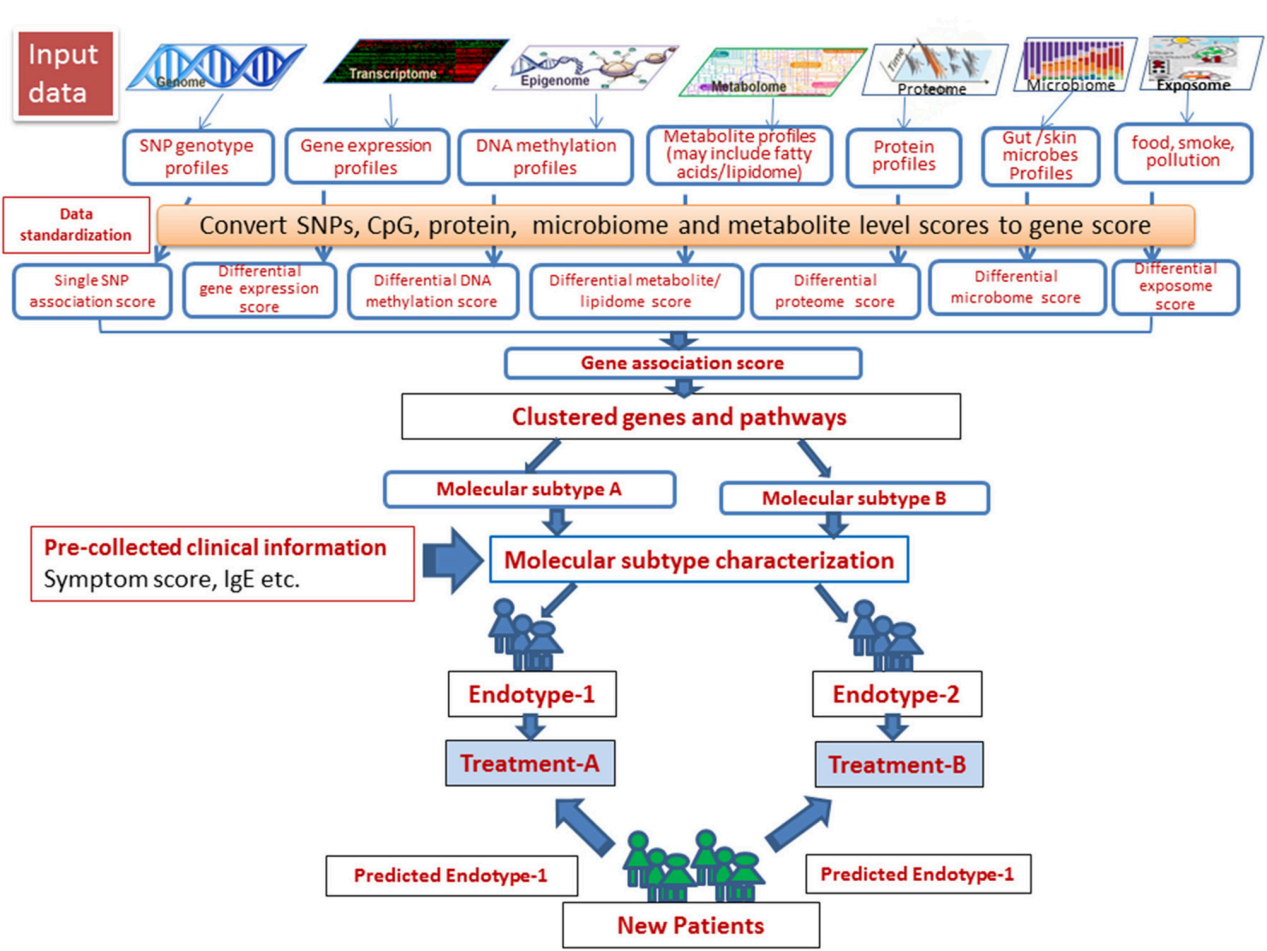
A schema for omics data integration to generate molecular subtypes for personalized medicine. With recent high throughput technologies, a wide range of omics level data has been generated. Omics datasets can be stored in publicly accessible databases that are continuously fed with curated data derived from omics-level experiments and scored to integrate using appropriate statistical tools. Next, the samples can be resolved into groups using integrated scores to identify molecular subtypes, which after associating with clinical phenotypes, can be used to designate endotypes. Endotype-specific genes/pathways can be useful for rational designing of treatment strategies.

Steps involved in a successful identification of disease subtypes and integration of multi-omics data and associated clinical outcomes measures have been described by Roger et al. (9). In such studies, patients were clustered using different omics-level data (exome sequences, transcriptome, and proteome data), and the results were integrated with clinical data to effectively characterize complex disease subtypes. The challenges with this “molecular-data-first” approach, however, need to be carefully addressed. In the case of AD, this model is largely impeded by limited or lack of information at multiple omics levels of particular clinical phenotypes included in the studies conducted thus far. We created a schema for omics integration in AD (Figure 4). Omics datasets can be obtained from publicly accessible databases that are continuously updated with curated data primarily derived from omics-level experiments. SNP set association scores for each gene can be computed on the basis of the SNPs assigned to the respective gene. Differential gene expression, DNA methylation and metabolite (including fatty acids for lipidome) scores can be computed for each gene from gene expression, DNA methylation and metabolite profiles. The gene association scores integrate evidences from expression, methylation, metabolite signature, and the genotype signature for each gene. Finally, the pathway/ network association test can identify gene sets associated with samples of a single endotype by integrating evidence across genes in the gene sets. This can identify molecular subtypes, which after associating with clinical phenotypes, can be used to designate endotypes. Endotype-specific genes and pathways can be useful for rational designing of treatment strategies.

## Omics-Wide Integration: Recent Advances and Remaining Challenges

The pathogenesis of AD has been attributed to adaptive immune abnormalities, with dysregulated Th1/Th2/Th22 response, IgE production, chemokine signaling, and dendritic cell recruitment, resulting in the itch and inflammatory dermatosis (208). It is increasingly evident that inflammation in AD results initially from inherited and acquired insults that converge to drive structural and functional alterations, followed by immune system activation and compromised skin-barrier homeostasis. This cycle has been designated as the “outside-inside-outside” model of AD (208). Th2 cytokines can down-regulate epidermal barrier protein, which can enhance antigen/allergen exposure and thereby increase inflammation. In spite of evolving models to explain etiology being proposed, the management of AD is primarily aimed at controlling inflammation and itch. The underlying mechanism of early emollient use in preventing AD is still not completely understood (209). In addition to Th2 activation, AD is probably much more heterogeneous, with additional activation of Th1, Th22, and Th17/IL-23 cytokine pathways depending on AD subtype and endotype (210). FLG mutation represents an early onset AD subtype with more severity. Among two other relatively clear phenotypes, intrinsic AD (normal IgE level) shows significantly elevated expression of IL-17, IL-23, IL-22 cytokines associated with S100As, elafin/PI3, CCL20 expression by keratinocytes (thus resembling psoriasis), whereas extrinsic AD (high IgE level) shows similar expression levels of Th2 cytokines (29). Although cytokine-targeted dupilimab therapy shows efficacy in both intrinsic and extrinsic AD, the exact mechanism is not completely clear in the case of intrinsic AD. Contrasting clinical, immunologic and histochemical features of AD and psoriasis have been elaborated elsewhere (211). Both lesional and non-lesional AD can be associated with epidermal barrier defects (as demonstrated by higher TEWL), with or without specific IgE. In molecular terms, psoriasis has been considered as a TH17/IL23–skewed disease, whereas AD has been considered a TH2-centered disease, both sharing TH22 components. However, Martel et al. demonstrated that the transcriptomic signature of intrinsic AD is more similar to psoriasis than to extrinsic AD (152). However, the TH17 and TH22 pathways along with expression of S100A and innate immune genes by keratinocytes are less activated in patients with AD compared to psoriasis (212). In addition to intrinsic AD, results involving Asian AD subjects (mostly extrinsic), showed an induction of the TH17 and TH22 axes (28). Taken together, these studies demonstrate there are molecular subtypes of AD that demonstrate features common with psoriasis, but does not conclusively indicate that they are the subtypes of psoriasis. Nevertheless, molecular subtyping of AD is very important in personalized treatment and results from ongoing clinical trials targeting TH1, TH2, TH17, and TH22 pathways will continue to dissect the patho-mechanisms associated to this complex condition.

The methods to analyze patient omics data into molecular subtypes can be adapted to fit the type and volume of data, leading to characterization of unique and shared pathways for molecular subtyping. Next, standard statistical models can be implemented for connecting molecular subtypes with clinical phenotypes. This approach previously led to successful identification of complex disease endotypes, understanding prognosis and appropriately targeting treatments (9). However, it has also been mentioned that since new patients may often have limited data compared to the patient group used to generate the molecular subtypes, endotyping needs to be based on simpler, easily obtainable data and preferably connected to stable biomarkers. Finally, a significant challenge for connecting multi-omics to complex disease endotypes is the availability of high quality phenotypic data. In case of AD, not all clinically relevant parameters (such as TEWL), are available for omics datasets. It is therefore important to collect as much phenotypic data as possible to correlate with different omics layers.

## Omics-wide Integration: Analytical Challenges and Opportunities

Big data has been defined as “large volumes of high velocity, complex, and variable data that require advanced techniques and technologies to enable the capture, storage, distribution, management and analysis of the information” (204, 213). Big data has led to new data warehouses and technologies including cloud-based computing with user-friendly input and output interfaces. Big data from real-time physiologic and environmental data connected to patients omics data can uncover novel pathways and processes related to complex disease etiology in the future (204). However, there are several challenges to integrate “big omics data.” Some of these challenges include problems with the data being scattered in several annotation databases and thereby a lack of a unified portal for data annotation and analytics. In addition, integrating all types of omics data (such the datasets generated from genomic, epigenomic, transcriptomic, proteomic, interactomic, metabolomic studies) are difficult due to non-linearity of the data. Seamless downstream analysis and prediction of actionable insight require multiple, disparate tools and manual interventions. In addition, emerging clinical data sources are typically less structured, since they were designed to serve a different purpose (e.g., clinical care and billing). Current integrative analyses are primarily focused on the combination of omics data from the same source material, such as tumor, human brain or blood samples representing one level of regulation. However, the integration of data from different levels of regulation (i.e., blood and tumor) or different patient samples may also reveal important knowledge about the hierarchy of the human genetic architecture.

High throughput technologies are becoming increasingly efficient and affordable. Moreover, efficient use of publicly available datasets and novel bioinformatic-based tools it can be possible to identify interactive networks of genes, transcripts, proteins and metabolites providing a framework for data integration useful to detect, rank and predict functional variants linked with AD. The rationale is that by integrating different omics data, we can learn more about genotype-phenotype relationships than would not be possible otherwise. Our ability to measure millions of data points for every single omics experiment makes hypothesis generation dependent on advanced computational approaches. Machine learning (ML) techniques are among the most widely used approaches to address this problem (214). ML represents computer algorithms that can learn from existing data and use that knowledge to make predictions from new data such as distinguishing between disease states on the basis of gene expression or methylation profiles of samples. ML algorithms can improve the accuracy of prediction over conventional regression models by capturing complex, non-linear relationships in multi-omics and clinical data. The algorithms used in ML include data mining and cluster approaches of supervised or unsupervised nature (215).

## Conclusion and Future Direction

The power of the multi-omics approach in personalized and predictive medicine for complex diseases has recently been emphasized by several authors (205, 216). Despite increased research efforts in recent years, most omics-level data concerns only genomics and transcriptomics in AD. There are some novel proteomic (in serum) and lipidomic (in sweat samples) studies showing great promise toward identifying AD endotypes. Using multi-omics analysis, we showed that previously suspected genes, such as *FLG* and *SERPINB3*, were associated with AD among multiple omics types. At the pathway level, macrophage, endothelial cell/ ET-1, Th1/Th2, NFκB, and fibroblast activation pathways were overrepresented in AD from multiple omics level results. Thus, integrated multi-omics data provide new biological insights, as well as confirm previously reported AD-associated genes and pathways. The relative importance of one or more pathways in individual patients might be helpful in establishing endotypes. It should be noted that not all biomarkers for identifying endotypes can be visible by interrogating one single omics layer; thus, more studies are required to unravel multi-layered omics data in AD. Because most of the advanced data integration tools mentioned above are to be used to analyze multidimensional omics data obtained from the same set of samples, future studies should be directed to consistently obtaining multilevel (e.g., genomic, transcriptomic, proteomic) data from AD samples to obtain an integrated omics level picture of the disease and its endotypes. Despite the growing availability of genome-wide data in multiple omics types, so far, publicly available omics databases contain limited data related to AD. To address this limitation, data obtained from all omics-level experiments (e.g., epigenome, lipidome exposome, microbiome) should be deposited in appropriate public data repositories. Improved computational tools are needed to handle big data generated by high-throughput technology along with a patient's personalized exposure profile to achieve the promise of precision medicine in AD.

## Author Contributions

DG and TM wrote the manuscript and JB, GK, and MR contributed to write the manuscript.

### Conflict of Interest Statement

The authors declare that the research was conducted in the absence of any commercial or financial relationships that could be construed as a potential conflict of interest. The reviewer AA and handling Editor declared their shared affiliation at the time of review.
